# Wheat Bread Supplemented with Egg Albumin: Structural Features, and In Vitro Starch and Protein Digestibility

**DOI:** 10.1007/s11130-024-01283-7

**Published:** 2025-01-21

**Authors:** J. Rosas-Rivas, M. E. Rodríguez-Huezo, E. J. Vernon-Carter, J. Alvarez-Ramirez

**Affiliations:** 1 Departamento de Biotecnología, Universidad Autónoma Metropolitana-Iztapalapa, Apartado Postal 55-534, Iztapalapa, CDMX 09340 Mexico; 2 Departamento de Ingeniería Química y Bioquímica, Tecnológico Nacional de México, TESE de Ecatepec, Av. Tecnológico s/n esq. Av. Central, Col. Valle de Anáhuac, Ecatepec, Estado de México, C.P. 55210 Mexico; 3 Departamento de Ingeniería de Procesos e Hidráulica, Universidad Autónoma Metropolitana- Iztapalapa, Apartado Postal 55-534, Iztapalapa, CDMX 09340 Mexico

**Keywords:** Bread, Egg albumin, Digestibility, Starch, Protein

## Abstract

This study aimed to explore the effects of egg albumin protein addition (5, 15 and 20 g/100 g db) on the textural characteristics, as well as in the in vitro digestibility of protein and starch of wheat bread. Egg albumin addition resulted in smoother bread loaves as compared to traditional wheat bread. Reduced hardness and increased cohesiveness were correlated to the protein secondary structure, mainly with the content of β-sheets. The in vitro protein digestibility decreased with the albumin addition, suggesting the mediation of protein-starch interactions. The in vitro starch digestibility was also decreased, reflected in a huge decrease of the slowly digestible starch fraction, but with non-significant changes in the rapidly digestible starch fraction. The supplemented albumin can form a physical barrier around the starch granules, which hampers the access of the amylolytic enzymes to the starch chains. Overall, the results of this study suggest that the addition of egg albumin is a viable alternative for producing wheat bread with reduced glycemic index and improved nutritional properties.

**Supplementary Information:**

The online version contains supplementary material available at 10.1007/s11130-024-01283-7.

## Introduction

Wheat bread is the most important source of carbohydrates for food consumers in Western countries. Starch is the principal component, with a mean content of about 40–45 g/100 g bread. In its more basic preparation method, wheat bread comprises only flour, water, salt and a leavening agent to form dough, which is baked at about 180 ^o^C. The relatively high digestibility of the bread starch can be deemed as the main source of energy for human activity. However, in the increasing migration of people to cities and the accompanying lower physical activity, have result in human health drawbacks (metabolic syndrome) in recent decades. This has motivated the search and manufacture for healthier bread formulations that provide improved nutritional benefits and are acceptable to consumers. The production of healthy-functional bread with reduced starch digestibility and nutrient-dense ingredients has been explored [1].

In recent years the interest in protein-dense bread has led to its fortification with protein from diverse sources [2]. Phongthai et al. [3] found that rice flour bread enriched with rice bran protein concentrate improved the elastic modulus of its batters and the bread specific volume. Contrarily, Alzuwaid et al. [4] supplemented wheat bread with wheat bran protein concentrate, finding that the sensory and textural characteristics were affected negatively. Ferreyra et al. [5] reported that addition of 20% of whey protein concentrate improved the in vitro protein digestibility of wheat bread. Azeez et al. [6] found that supplementing wheat bread with cashew nut protein concentrates significantly increased the protein, ash, crude fiber, calcium, magnesium, iron, phosphorus and zinc contents, while carbohydrate value decreased. Qazi et al. [7, 8] explored the addition of proteins extracted from three different microalgae, finding that all of them scored low on the sensory properties of wheat bread. Montevecchi et al. [9] reported that protein extracted from microalgae improved the nutritional characteristics of wheat bread, but could imprint non-desired flavors. Pořízka et al. [10] showed that wheat bread incorporated with whey protein isolates had better consumer acceptance scores than when added wheat bran protein isolates, which produced an unacceptable bitter taste. Komeroski and Oliveira [11] reported that the addition of whey proteins modified the physical characteristics and improved the chemical composition of the wheat bread. But concentrations of ≥ 30% produced poorer texture characteristics.

Bread as one of the most common foods worldwide, can be fortified for helping to eliminate nutrients deficiencies, and for providing the sustainable health benefits of consumers. But fortification must be achieved with low-cost high-quality nutrient ingredients, or price increase of the fortified bread will render it inaccessible to the consumers strata most requiring it [12]. In this sense, egg albumin is a complete protein, containing all nine essential aminoacids. It is highly digestible, and has been shown to decrease malnutrition in underdeveloped countries, can help children gain height increase, and prevents the incidence of kwashiorkor [13]. Thus, egg albumin has been recognized as an important ingredient for ketosis diets [14]. Recently, Boev and Trubnikov [15] showed that egg albumin led to a decrease in alkalinity and moisture content.

Most of the research studies on the protein fortification of wheat bread have focused on physicochemical and sensory properties, but results on the impact of protein addition in the digestibility of starch and protein are still scarce. This work focuses on how the added egg albumin protein can interact with the wheat starch, and how the in vitro digestibility of starch and proteins of wheat bread is affected.

## Materials and Methods

The detailed description of the materials and the methods used in the study are provided in the accompanying supplementary material.

## Results and Discussion

### Dough Rheology

**Fig. 1:**
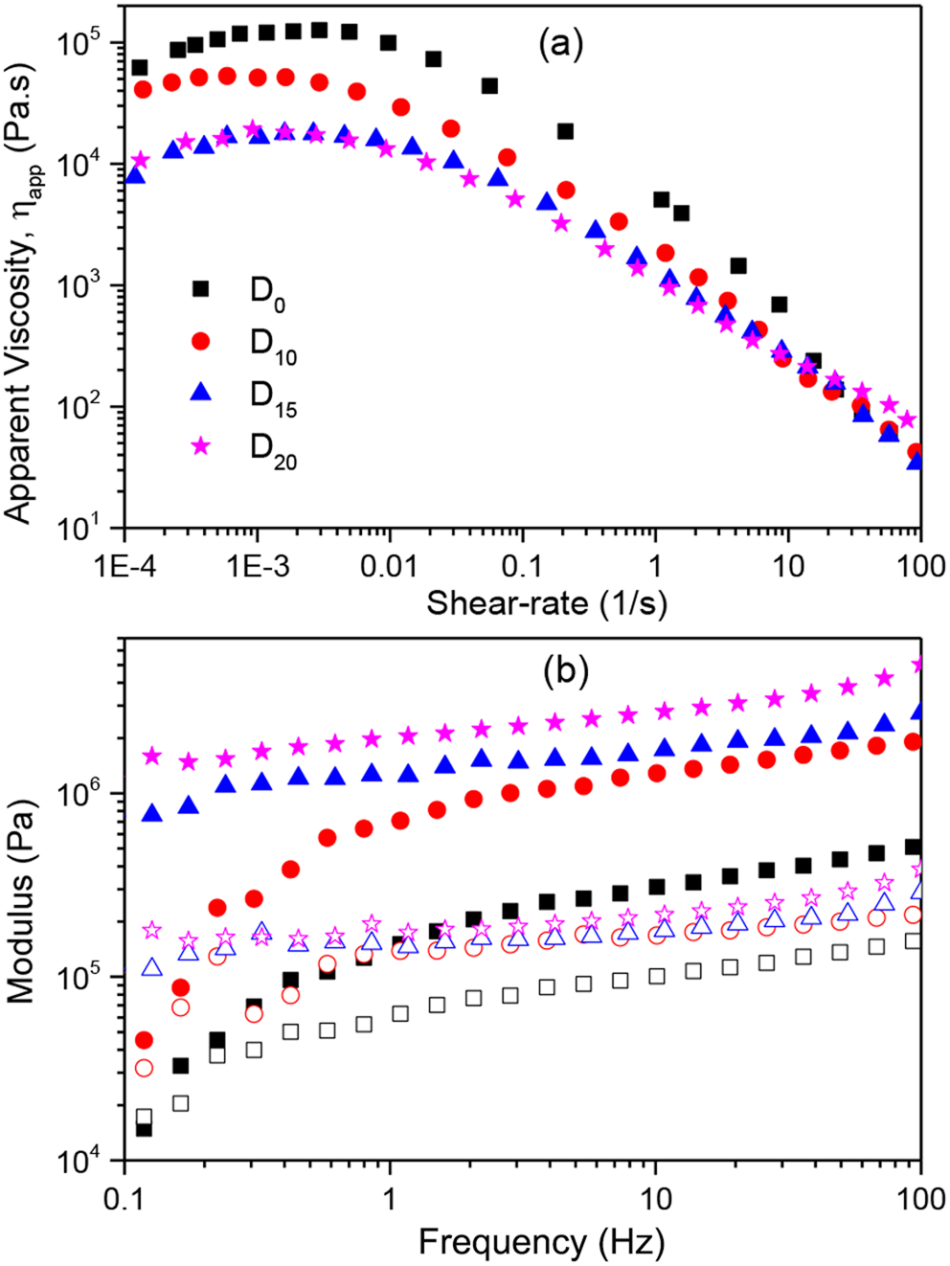
(**a**) Apparent viscosity and (**b**) storage (G’) and loss (G’’) moduli for doughs with different protein content. Full symbols denote G’ and empty symbols G’’

Dough rheology is an important issue in bread manufacturing. Figure 1.a presents the behavior of the apparent viscosity (η_app_) as a function of shear rate. For small shear rate values, up to about 0.001 s^− 1^, the dough behaves as a shear-thickening material. The viscosity increased due to the rearrangement of starch particles. For higher shear rate values, the behavior is consistent with a shear thinning pattern. The albumin addition decreased the dough apparent viscosity, suggesting that the additional protein tended to coat the starch granules impeding them to uptake water and expand. As a consequence, the apparent viscosity decreased as the smaller protein-covered starch granules flowed past each other in the changing shear rate flow field. In contrast, the loss and storage moduli increased with the albumin addition as it interacts with the starch granules giving way to gelled structures, which under non-destructive testing, exhibit enhanced rheological properties. Results in this line were reported by Paraskevopoulou et al. [16] for wheat flour-lupin protein isolate blends.

### Bread Texture

The bread texture parameters are presented in Table 1. Bread hardness, chewiness and elasticity decreased significantly decreased compared to the control bread (B0). Pietro-Morales et al. [17] found that there is not a general rule on how different added proteins impact the bread texture. For instance, soy, pea and faba protein isolates increased bread hardness, but maize germ and chia seed proteins can lead to a marked decrease. It was suggested that the different textures related to hardness are possibly due to water competition between protein and starch. Table 1 shows that albumin creates softer bread, although with decreased elasticity. In contrast, protein addition increased the bread cohesiveness, meaning that protein-enriched bread has a better disintegration resistance during mastication. Likewise, resilience also increased, suggesting that the protein addition can improve the acceptability of consumers [18]. Boev and Trubnikov [15] showed that egg albumin changed the bread porosity, moisture content and alkalinity. These indicators can be linked to the variations of the bread texture with the different egg albumin contents.

**Table 1:** Texture parameters of bread with different egg albumin content

Bread	Hardness (*N*)	Chewiness (mJ)	Elasticity (mm)	Cohesiveness (-)	Resilience (-)
B0	35.52 ± 1.92^a^	219.07 ± 8.73ª	10.65 ± 0.78ª	0.62 ± 0.03^b^	0.28 ± 0.01^b^
B10	18.53 ± 1.26^b^	52.07 ± 4.79^b^	4.07 ± 0.25^b^	0.83 ± 0.04ª	0.53 ± 0.02ª
B15	13.51 ± 1.75^c^	40.70 ± 3.96^b^	3.39 ± 0.19^c^	0.87 ± 0.04ª	0.54 ± 0.02ª
B20	14.32 ± 1.43^c^	48.87 ± 4.25^b^	3.34 ± 0.24^c^	0.81 ± 0.03ª	0.49 ± 0.02ª

Values are means ± standard error, of three replicates. Superscripts with different letters in same column indicate significant differences (*P* ≤ 0.05). BX denotes the bread formulation with “x” denoting the albumin addition (g/ 100 g flour db)

### FTIR Analysis

The molecular organization of proteins and starch in the bread was explored with FTIR analysis. The variation of the secondary structure distribution with the albumin addition is exhibited in Table 2. Albumin addition decreased the content of coils and increased the content of β-sheets. The content of random structures was only slightly modified. The gluten protein secondary structure, especially β-sheets, has been linked to wheat dough rheology and bread texture [19]. Negative.

**Table 2:** FTIR parameters obtained from numerical deconvolution

Bread	Coils (%)	Random (%)	β-sheets (%)	995/1022	1044/1022
B0	38.01 ± 1.23ª	32.64 ± 0.91ª	29.33 ± 0.78^c^	0.52 ± 0.02ª	0.42 ± 0.01ª
B10	39.08 ± 1.17ª	31.51 ± 0.85ª	29.40 ± 0.89^c^	0.44 ± 0.02^b^	0.39 ± 0.02^b^
B15	35.64 ± 1.04^b^	33.01 ± 0.87ª	31.34 ± 0.75^b^	0.39 ± 0.01^c^	0.37 ± 0.02b^c^
B20	35.33 ± 1.05^b^	29.32 ± 0.98^b^	35.34 ± 0.94ª	0.34 ± 0.02^d^	0.30 ± 0.02^d^

Values are means ± standard error, of three replicates. Superscripts with different letters in same column indicate significant differences (*P* ≤ 0.05). BX denotes the bread formulation with “x” denoting the albumin addition (g/ 100 g flour db)

correlations between β-sheets and hardness (-0.54, sig. 0.41) and elasticity (-0.55, sig. 0.48) were found. Likewise, positive correlations between β-sheets and cohesiveness (0.59, sig. 0.61) was detected. Table 2 also shows the FTIR intensity ratios 995/1022 and 1044/1022, which reflect the content of hydrated and ordered starch structures relative to amorphous structures. Both ratios decreased with the albumin content, suggesting an important interaction of added proteins with the starch chains. It seems that the presence of the albumin limited the hydration of starch granules and the formation of short-range ordered (e.g., double-helix) structures, due maybe to the formation of a physical barrier about the starch granules [20].

### Protein Digestibility

**Fig. 2:**
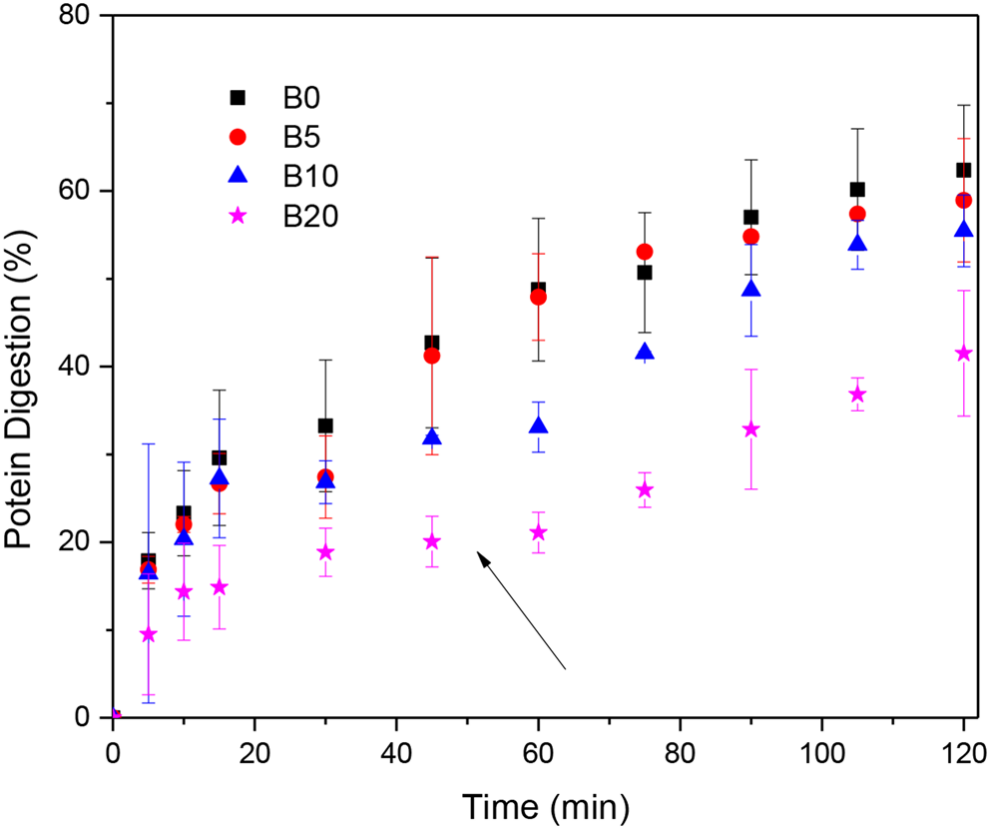
Kinetics of protein digestibility for bread (BX) with different albumin contents (X)

The kinetics of the *in vitro* protein digestibility is shown in Fig. 2. For the control bread B0, kinetics exhibit exponential-like behavior with a digestion time constant of about 40 min. This means that about 64% of the total digested protein was hydrolyzed in the first 40 min. The bread B5 exhibited a similar hydrolysis pattern, with a hydrolysis time constant of about 42 min. The further addition of protein changed the hydrolysis kinetics pattern. In the first stage, up to about 30 min, the protein hydrolysis showed an exponential-like behavior. However, this pattern does not hold for subsequent times as the hydrolysis kinetics exhibited plateau behavior for times 30–65 min as indicated by the arrow in Fig. 2. In this stage, the protein was scarcely hydrolyzed. For longer times, the hydrolysis kinetics retook the pattern already shown for relatively short times. The stacking of the in vitro hydrolysis for medium times suggests that the bread protein has two structures. Both gluten and albumin are mixed with the carbohydrates, forming a complex structure. A fraction of the proteins can be exposed for easy access to proteases, which result in proteins that are rapidly digested (within the first 20–25 min). A second fraction of the proteins is embedded in the starch matrix, hindering the accessibility to proteases. The disruption of the protein-carbohydrates matrix by effect of protein hydrolysis and water penetration results in a further access to protein hydrolysis. This fraction can be seen as a slowly digested protein fraction.

Protein digestibility (PD) was taken as protein hydrolysis after 120 min, which is the mean residence time of the chyme in the small intestine. Figure 2 shows that the protein addition led to a significant decrease (*p* < 0.05) of the protein digestibility, and the results are summarized in Table 3. The in vitro digestibility decreased from about 62.64% for the control bread B0 to about 43.12% for the bread (B20) with 20% albumin content. Lu et al. [21] found that endogenous and exogenous proteins form a coat over starch granules inhibiting their expansion, and precluding the accessibility of digestive enzymes, delaying starch digestion (and protein digestion as found in this work).

**Table 3:** In vitro digestibility results of protein and starch

Bread	PD (g/100 g protein)	RDS (g/100 g starch)	SDS (g/100 g starch)	RS (g/100 g starch)
B0	62.64 ± 1.12^a^	23.91 ± 0.78^b^	66.26 ± 1.23ª	9.83 ± 0.91^d^
B10	59.32 ± 1.42^b^	25.68 ± 0.25ª	58.06 ± 1.17^b^	16.26 ± 0.85^c^
B15	55.49 ± 1.38^c^	26.39 ± 0.19ª	35.18 ± 1.04^c^	38.43 ± 0.87^b^
B20	44.12 ± 1.43^d^	24.26 ± 0.24^b^	27.31 ± 1.05^d^	48.43 ± 0.98ª

Values are means ± standard error, of three replicates. Superscripts with different letters in same column indicate significant differences (*P* ≤ 0.05). BX denotes the bread formulation with “x” denoting the albumin addition (g/ 100 g flour db)

### Starch Digestibility

**Fig. 3:**
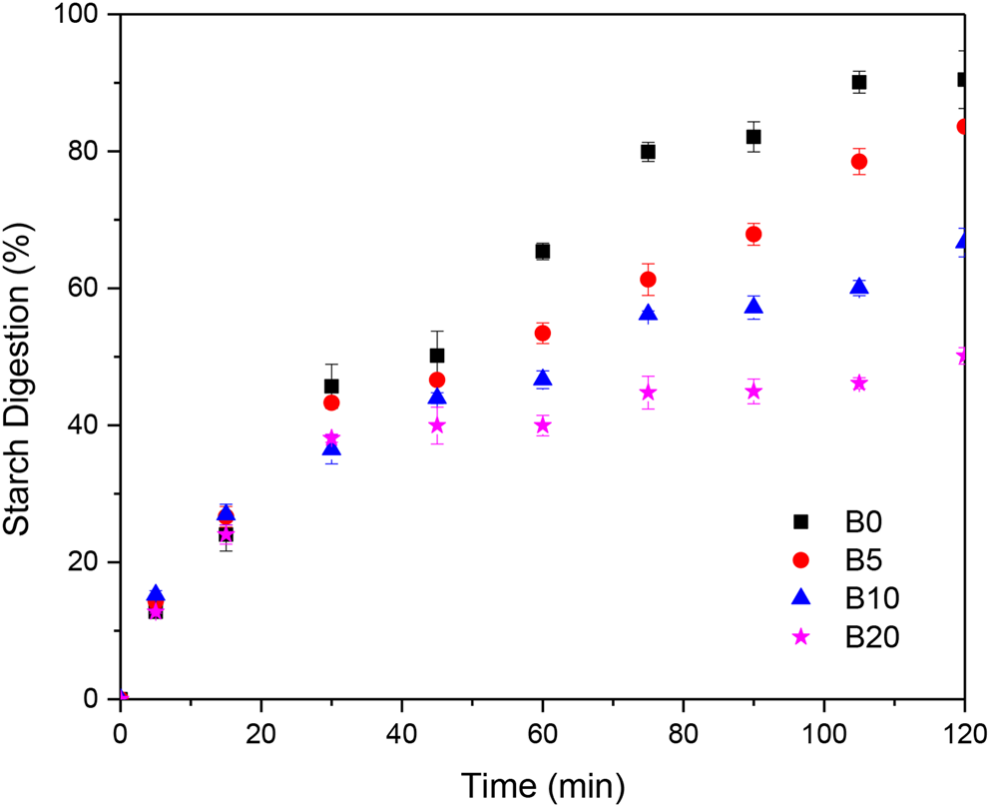
Kinetics of starch hydrolysis for bread with different albumin contents

Figure 3 presents the hydrolysis kinetics of starch of the bread formulations. For the control bread (B0), the hydrolysis kinetics exhibits an exponential-like behavior, with a characteristic time constant of about 52 min. However, this pattern is not exhibited by the bread added with albumin. Like the protein hydrolysis kinetics (Fig. 2), the starch hydrolysis displays three stages. In the first stage, the hydrolysis kinetics follows an exponential pattern lasting up to 40 min. Afterwards, the starch hydrolysis exhibits a sluggish stage caused by hampered access to the starch chains by the amylolytic enzymes. For times longer than about 60 min, the exponential-like pattern is restored due maybe to the disruption of the bread matrix, causing easy access to hydrolytic enzymes. The starch and protein hydrolysis kinetics suggest that the added albumin modified the bread structure by forming a complex network of protein structures surrounding the starch granules. The in vitro starch digestibility results are summarized in Table 3. The RDS was scarcely affected by the addition of albumin. In contrast, the SDS content exhibited a huge decrease as albumin addition was increased to 20 g/100 g. RDS is linked to the extent of gelatinized starch, while SDS is linked to the hydrolysis of starch granules. The decrease of the SDS with the concomitant increase of the non-digested starch suggests that the albumin acted as a physical barrier that shielded the starch structure against the action of the amylolytic enzymes. The results in Table 3 are in line with recent studies reporting that the addition of protein from different sources can reduce the digestibility of starch [22]. Lu et al. [23] postulated that protein inhibited starch granule swelling in starch/protein blends during cooking.

## Conclusions

Exogeneous addition of albumin modifies the textural characteristics and improves the nutritional quality of wheat bread, and produced a softer bread loaf. The albumin addition decreased the in vitro digestibility of starch by reducing the slowly digestible starch fraction whilst leaving unchanged the rapidly digestible starch fraction. Thus, a reduction of the glycaemic index was achieved as less starch is converted in sugars. However, it has the drawback of limiting the energetic contribution of wheat bread via slowly digestible starch as this fraction acts as a modulator of the glucose levels in plasma. On the other hand, although the amount of protein is increased by the albumin addition, the in vitro protein digestibility was decreased. Therefore, the digested protein is not aligned with the added protein. There is a trade-off between starch and protein digestibility since reduced digestibility of the former and increased digestibility of the latter are desirable in a presumably healthy bread. Overall, exogeneous protein addition offers a framework to the formulation of wheat bread with reduced starch digestibility and increased protein content.

## Electronic supplementary material

Below is the link to the electronic supplementary material.

Supplementary Material 1

## Data Availability

Data is available upon request to the corresponding author.

## Abbreviations

BE: β-sheet structures
CO: Coils Structures
FTIR: Fourier Transform Infrared Spectroscopy
PD: Protein Digestibility
RA: Random Secondary Structures
RDS: Rapidly Digestible Starch
SDS: Slowly Digestible Starch
