# Supplementary material for: Wheat Bread Supplemented with Egg Albumin: Structural Features, and In Vitro Starch and Protein Digestibility
Source: Plant Foods Hum Nutr. 2025 Jan 21;80(1):41. doi: 10.1007/s11130-024-01283-7 (PMC11750911; doi:10.1007/s11130-024-01283-7)
Supplement: Supplementary file 1 — Supplementary Material 1 [file 11130_2024_1283_MOESM1_ESM.docx]

**Supplementary Material**

**Wheat Bread Supplemented with Egg Albumin: Structural Features, and In Vitro Starch and Protein Digestibility**

J. Rosas-Rivas^1^, M.E. Rodríguez-Huezo^2^, E.J. Vernon-Carter^3,*^ and J. Alvarez-Ramirez^3^

^1^Departamento de Biotecnología, Universidad Autónoma Metropolitana-Iztapalapa, Apartado Postal 55-534, Iztapalapa, CDMX, 09340 México

^2^Tecnológico Nacional de México, TESE de Ecatepec, Departamento de Ingeniería Química y Bioquímica, Av. Tecnológico s/n esq. Av. Central, Col. Valle de Anáhuac, Ecatepec, C.P. 55210, Estado de México, México

**Materials and Methods**

**Materials.** Wheat flour (Tres Estrellas®, 73 g carbohydrates, 11 g protein, 1 g lipids, 2 g dietary fiber and 0.0016 g ashes per 100 g flour), sugar, salt, baking dry yeast (TradiPan S.A. de C.V., CDMX, Mexico) were purchased at a local supermarket (Walmart, CDMX, Mexico). Food-grade egg albumin was purchased from Huevo San Juan S.A. de C.V. (San Juan de los Lagos, State of Jalisco, Mexico), with composition according to the manufacturer: 0.05 g lipids, carbohydrate 9.95 g, 5.0 g total fiber, 80.g protein per 100 g db. All reagents used were analytical grade. Distilled water was used in all experiments.

**Bread preparation.** Bread was prepared according to the procedure reported by Gonzalez et al. [1]. The ingredients were wheat flour (100 g), water (60 g), sugar (3 g), salt (1.5 g), dry yeast (4.5 g) and egg albumin (0, 10, 15 and 20% wt egg albumin/wt wheat flour). Bread components were mixed for 10 min. (Laboratory Spiral Mixer, SP-800-J Alpha Simet Group, Germany). Dough was allowed to stand for 20 min in silicone containers placed on a static convection oven (Rational AG, Landsberg, Germany) at 37 °C and 20% relative humidity. Afterwards, the dough was divided into portions of 25 g and baked in a convection oven Oster ® for 20 min. at various temperatures 180 °C. After baking, bread was allowed to cool down at room temperature and stored in hermetic bags. Doughs and bread were coded as D_X_ and B_X_, respectively, where the subindex “x” denotes albumin content.

**Dough rheology.** Oscillatory strain and rotational measurements of the different dough formulations were carried out in a Physica MCR 300 rheometer. The measuring geometry consisted of serrated plates (gap of 0.5 mm), with 50 mm diameter. The apparent viscosity curves were obtained by varying the shear rate in a logarithmic ramp from 0.0001 to 100 s^-1^. Amplitude sweeps were obtained for 0.1-100% at 1.0 Hz. The storage (G’) and the loss (G’’) moduli were recorded and analyzed with the rheometer software (US200/32 V2.50). Measurements were done at 25 °C and temperature was controlled with a Peltier system.

**Fourier transform infrared spectroscopy (FTIR).** Infrared spectra were determined with an FTIR spectrophotometer (Frontier, Perkin Elmer®), equipped with a universal attenuated total reflectance accessory. Approx. 10 mg of dough or bread were brought into contact with the diamond crystal using a force setting of 60 units, and absorbance wavelengths from 4000 to 400 cm^-1^ were used, with a resolution of 40 cm^-1^ [2]. A numerical deconvolution procedure with Gaussian function was carried out to obtain individual contributions for distinctive bands.

**Texture.** Texture profile analysis (TPA) was performed 30 min after baking process, using a Brookfield® CT3-4500 texturometer (AMETEK Brookfield, Middleborough, MA, USA) equipped with a T4/1000 cylinder. Bread loaves were compressed up to 20% of their original height. The analysis consisted of two compression cycles using a 5 g trigger load and a speed of 10 mm/s. Three loaves from each type of formulation were analyzed. The parameters of elasticity, adhesiveness, cohesiveness, hardness, resilience and chewiness were evaluated and were obtained with the equipment software.

**In Vitro Digestibility. P**rotein digestibility (PD) was determined as reported by [3]. Porcine pancreatic trypsin (Type IX, 15,310 units/mg protein), bovine b pancreatic chymotrypsin (Type II, 48 units/g solid), porcine intestinal peptidase (P-7500, 115 units/mg solid) and bacterial protease (Type XIV, 4.4 units/mg solid) were used for enzymatic digestion. Crumbled bread (~ 25 mg) samples were added to 10 mL of distilled water and homogenized (Ultra-Turrax® T50 basic IKA Works, Inc., 164 Wilmington, DE, USA), at 6,000 rpm for 1.5 min, using an ice bath for avoiding temperature increase. Afterwards, the pH of the obtained mixture was adjusted to 8.0 with 1 N NaOH. One mL of enzyme aqueous solution (1.58 mg of trypsin, 3.65 mg of chymotrypsin and 0.45 mg of peptidase) was added to the protein sample and digestion was allowed to proceed for 10 min at 37 °C. After addition of 1 mL (1.48 mg) of bacterial protease solution, the digestion was continued for 9 min at 55 °C. The pH value was registered and used to estimate the PD according to the following expression:

$PD\left( \% \right)=234.84-22.56pH$, where $pH$ is the pH of the suspension [4].

The i*n vitro* starch digestion was determined following the methodology by Englyst et al. [5]. Total starch (200 mg) sample isolated from bread crumb samples was incubated with pancreatin from porcine pancreas (300 IU/mL, P1750) and amyloglucosidase (95 IU/mL, A7095) enzymes. A temperature of 37 °C and an incubation time of 120 min were used for both enzyme treatments. After hydrolysis for 20 or 120 min, the tubes were boiled for 10 min to deactivate the enzyme and terminate the reaction and centrifuged at 5000 ×g for 10 min. The glucose content in the supernatant was measured by the 3,5 dinitrosalicylic acid (DNS) method. The percentage of hydrolyzed starch was calculated by multiplying a factor of 0.9 to change values for glucose to starch. The percentage of the different hydrolyzed starch fractions were calculated with the following equations:

RDS (%) = (G_20_ × 0.9/W) × 100; SDS (%) = [(G_120_−G_20_) × 0.9/W] × 100; and RS (%) = 100−RDS−SDS, where G20 and G120 are glucose content released after 20 and 120 min, respectively; and W is the starch weight (mg) used for each test.

**References**

1. González M, Vernon-Carter EJ, Alvarez-Ramirez J, Carrera-Tarela Y (2021) Effects of dry heat treatment temperature on the structure of wheat flour and starch in vitro digestibility of bread. Int J Biol Macromol 166: 1439-1447. https://doi.org/10.1016/j.ijbiomac.2020.11.023
2. Martínez-Velasco A, Lobato-Calleros C et al (2018) High intensity ultrasound treatment of faba bean (*Vicia faba* L.) protein: Effect on surface properties, foaming ability and structural changes. Ultrason Sonochem 44: 97-105. <https://doi.org/10.1016/j.ultsonch.2018.02.007>
3. Elsohaimy S A, Refaay T M, Zaytoun M A M (2015) Physicochemical and functional properties of quinoa protein isolate. Ann Agric Sci 60: 297-305. <https://doi.org/10.1016/j.aoas.2015.10.007>
4. Martínez-Velasco A, Alvarez-Ramirez J et al (2018) Effect of the preparation method and storage time on the in vitro protein digestibility of maize tortillas. J Cereal Sci 84: 7-12. <https://doi.org/10.1016/j.jcs.2018.09.016>
5. Englyst HN, Kingman SM, Cummings JH (1992) Classiﬁcation and measurement of nutritionally important starch fractions. Eur J Clin Nutr 46: S33–S50.
